# Effects of Self-focused Augmented Reality on Health Perceptions During the COVID-19 Pandemic: A Web-Based Between-Subject Experiment

**DOI:** 10.2196/26963

**Published:** 2021-06-29

**Authors:** Ayanna Seals, Monsurat Olaosebikan, Jennifer Otiono, Orit Shaer, Oded Nov

**Affiliations:** 1 New York University Brooklyn, NY United States; 2 Tufts University Medford, MA United States; 3 Wellesley College Wellesley, MA United States; 4 Department of Technology Management and Innovation Tandon School of Engineering New York University Brooklyn, NY United States

**Keywords:** COVID-19, health behavior, augmented reality, self-focused attention, vicarious reinforcement, human-computer interactions, hand hygiene, perception

## Abstract

**Background:**

Self-focused augmented reality (AR) technologies are growing in popularity and present an opportunity to address health communication and behavior change challenges.

**Objective:**

We aimed to examine the impact of self-focused AR and vicarious reinforcement on psychological predictors of behavior change during the COVID-19 pandemic. In addition, our study included measures of fear and message minimization to assess potential adverse reactions to the design interventions.

**Methods:**

A between-subjects web-based experiment was conducted to compare the health perceptions of participants in self-focused AR and vicarious reinforcement design conditions to those in a control condition. Participants were randomly assigned to the control group or to an intervention condition (ie, self-focused AR, reinforcement, self-focus AR × reinforcement, and avatar).

**Results:**

A total of 335 participants were included in the analysis. We found that participants who experienced self-focused AR and vicarious reinforcement scored higher in perceived threat severity (*P*=.03) and susceptibility (*P*=.01) when compared to the control. A significant indirect effect of self-focused AR and vicarious reinforcement on intention was found with perceived threat severity as a mediator (b=.06, 95% CI 0.02-0.12, SE .02). Self-focused AR and vicarious reinforcement did not result in higher levels of fear (*P*=.32) or message minimization (*P*=.42) when compared to the control.

**Conclusions:**

Augmenting one’s reflection with vicarious reinforcement may be an effective strategy for health communication designers. While our study’s results did not show adverse effects in regard to fear and message minimization, utilization of self-focused AR as a health communication strategy should be done with care due to the possible adverse effects of heightened levels of fear.

## Introduction

### Overview

With self-focused augmented reality (AR) usage increasing in recent years [1], the utilization of this technology has a potential in addressing health communication and behavior interventions challenges. Whereas AR technology layers digital content onto the real world [2], self-focused AR visually augments the self, layering digital content onto the self. One technology enabling self-focused AR is video filters, which superimpose computer-generated content onto a user using their web or smartphone camera (eg, Snapchat Lenses [3] and AR effects on Instagram [4]). For example, Snapchat’s Time Machine AR lens morphs a user’s reflection to display what the user might look like at different ages. Smart mirrors, another self-focused AR technology, combine digital screens (eg, an LCD [liquid-crystal display] monitor) with semitransparent glass. A smart mirror looks and functions similarly to a traditional mirror but with digital content displayed in the foreground [5]. Real-world applications of smart mirrors are on display in vehicles [6], dressing rooms [7], and home gyms [8]. For example, the MIRROR home gym [8] displays fitness instruction layered on top of the user’s reflection. Both AR video filters and smart mirrors provide new opportunities for displaying health behavior communication to the public.

In response to the recent increase in self-focused AR usage [1,6-8], we investigated the potential impact of self-focused AR within the health domain. For behavior change researchers, the effect of layering health threats along with mitigative behaviors and their results on top of a user may be of particular interest. For example, would using AR to layer a set of very healthy teeth due to good oral hygiene on dental patients impact their behavior? Could using AR in rearview mirrors to overlay scratches and bruises on top of drivers and riders encourage seat belt usage? We now have self-focused AR technologies in the hands of millions, presenting the opportunity to visually show individuals the impact of their decisions before they make them.

Research in psychology suggests that heightening self-focused attention (manipulated by using a mirror or video camera) has implications for perception, affective experiences (emotions, feelings, and moods), and behavior [9]. Objective self-awareness occurs when an individual places attention on themself, viewing themself as a social object. Objective self-awareness theory [10] posits that self-focused attention heightens the awareness of the gap between one’s perceived “real self” and “ideal self,” resulting in negative affect. For example, if one desires good health and believes that exercise is vital to maintain one’s health and yet does not exercise, heightening objective self-awareness will likely result in negative emotions. The increased negative affect resulting from the awareness of discrepancies leads to either (1) the avoidance of self-focused attention and the discrepancy or (2) actions to reduce the discrepancy [10]. This theory suggests that self-focused AR might impact behavior. We investigated which perceptions could be involved when individuals experience self-focused AR within a health context.

Research prototypes have explored self-focus [11] and self-focused AR [5,12-14] technologies for health behavior change. However, few have investigated how the design of interventions that aim to increase self-focused attention might impact health perceptions and emotions. Similarly, prior studies did not consider the potential of combining self-focused AR with vicarious reinforcement, that is, reinforcement from observing others’ behavior and the results of those actions. This paper draws upon insights from objective self-awareness theory [2] and social cognitive theory [15] to inform hypotheses about the relationships between predictors of health behavior change and self-focused AR.

We present findings from an online experiment on the impact of combining self-focused AR with vicarious reinforcement, visualizing the cause and effect of risk-mitigating behavior layered onto one’s reflection. Our study took place during the COVID-19 pandemic, focusing on hand hygiene behavior as an effective measure against pathogen transmission [16,17]. We discuss the implications of the results in light of the public health emergency, addressing the following research question: how does reinforcement in self-focused AR impact health perceptions during a pandemic?

### Background

Various health behavior change models [18,19] highlight the roles of predictors of intentions such as risk perceptions (perceived threat severity and threat susceptibility) and outcome expectancy. Drawing on objective self-awareness theory and social cognitive theory, we postulated that health behavior change–themed self-focused AR could impact these predictors of intention.

Research suggests that self-focused attention can result in action consistency with behavioral standards [9]. Objective self-awareness theory posits that self-focused attention will result in negative affect through the increased awareness of contradicting beliefs about one’s self or discrepancies between belief and behavior [10]. If negative affect is experienced, and one does not avoid the self-focused attention, they will attempt to reduce the discrepancy to reduce the negative affect, such as by changing their behavior. To further illustrate this, recall the individual from the example earlier who values their health and believes that exercise is vital for their health but does not exercise. According to objective self-awareness theory, an increase in self-focused attention would result in an attempt to reduce the discrepancy, which may result in exercise behavior. We propose that when increasing self-focused attention in the context of a health threat, especially during a pandemic, that the negative affect experienced will include fear and will increase to levels higher than if self-focused attention was not activated.

Research suggests that fear may play a large role in health behavior, especially during public health emergencies such as the current pandemic. Harper et al [20] found COVID-19 fear scores to be a positive predictor of behavior change. Fear may also impact behavior as it relates to risk perception. Risk perception, an individual’s perceived susceptibility to or severity of a threat, is included in many health behavior change theories [21]. Li [22] found perceived threat (measured by averaging threat susceptibility and severity) to be a positive predictor of fear. Affective factors are believed to play a role in the formation of risk perception [23]. While risk perceptions can increase fear, fear has also been found to induce higher risk perceptions [24]. Self-focused AR content layering onto the body increases the sense of spatial presence (ie, the object “being there”), potentially heightening fear if the object is threatening. Due to the combination of self-focus and spatial presence, we suggest that health threat–related self-focused AR may impact levels of fear, perceived threat severity, and perceived threat susceptibility.

It is important to consider the potential negative impact of heightening fear and risk perceptions. While Harper et al [20] found increased fear to be associated with higher behavioral adherence, they also found fear to be correlated with decreased physical and environmental quality of life and warned about mental health implications. Fear can also have an adverse effect on behavior. The Extended Parallel Process Model (EPPM) [25] outlines the importance of a balance of fear and efficacy for health communication campaigns to be effective. While fear can be a motivator for behavior, where the fear/efficacy balance is disrupted, individuals may use cognitive defense mechanisms instead of behavior as a means of fear control. In this case, not only would the behavior change method be ineffective, but it could result in the adverse effect of prompting the development of these defense mechanisms. Based on the EPPM, Li [22] tested a model for protective behaviors during a public health emergency with a study during the Ebola outbreak of 2014. Fear controls measured included negative reactance to messages, message minimizing, and defensive avoidance. Li [22] found perceived threat to have a significant effect on fear and fear controls but did not find self-efficacy to be a successful moderator of that relationship. This suggests that although fear may be an effective strategy to encourage health behavior adherence, certain levels of fear may lead individuals to minimize health behavior messaging to control their fear instead of engaging in behavior change. We take these findings and the EPPM into consideration, as our study directly layers a health threat onto participants, which could result in excessive levels of fear triggering adverse fear control mechanisms. We expected self-focused AR, in the form of AR video filters, to heighten both fear and fear control mechanisms when displaying a health threat.

Our study investigated the impact of vicarious reinforcement outcome expectancy when combined with self-focused AR. Research suggests that outcome expectancy mediates the impact of self-focused attention on behavior. For someone who has been made aware of a discrepancy between “actual state” and “desired state,” if they don’t believe a suggested behavior change will result in the “desire state,” they are more likely to change the “desired state” [9]. When the “desired state” is health related, this can have adverse implications. Outcome expectancy can be impacted by experiencing vicarious reinforcement. Vicarious reinforcement occurs when a reinforcing effect for an individual takes place by observing others’ behavior and the results of their actions [26]. Bandura et al [27] found that children who were exposed to media displaying aggressive behavior that was rewarded showed more imitative aggressive behavior than those who saw aggressive behavior that was punished. Bandura’s [15] social cognitive theory details how behaviors can be formed by observing a model engage in a behavior. Bandura suggests that due to limited contact with physical and social environments, people rely largely on vicarious experiences to form their idea of reality. In our study, vicarious reinforcement consists of visual representations of pathogens (ie, germs), which are made visible on avatar hands. As a hand hygiene animation plays, covering all the steps of proper handwashing, these pathogens disappear from the avatar’s hands. We predicted that this experience would heighten one’s perception of outcome expectancy. Given that health behavior models, such as The Health Action Process Approach [18] attribute outcome expectancies to the formation of intention, we found this valuable to investigate in our study. We proposed that the vicarious experience described above would directly affect outcome expectancy.

### Related Work

While studies combining self-focused attention and vicarious reinforcement have yet to see much direct utilization in human-computer interaction research, a few studies on health smart mirrors [5,12,13], self-representation [28], and spatial presence [14] suggest an impact of self-focused attention on perceptions and behavior.

While applications of smart mirrors for health care are limited, exploratory research prototypes have shown their potential to detect emotional states, monitor physiological parameters, and encourage behavior change. The Wize Mirror [12] encouraged users to improve their lifestyle to mitigate cardiometabolic risk assessed by tracking physical face signs )eg, skin color, subcutaneous fat, facial expressions). Medical Mirror [5] utilized computer vision and advanced signal processing within a smart mirror design to encourage people to keep track of their vital signs regularly. Fit Mirror [13] increased user’s motivation, happiness, and fitness for the day by integrating exercising and challenging others into their morning routine. Although the rise of smart mirrors has resulted in studies exploring the use of these devices in health care, there is a lack of research investigating how self-focus specifically plays a role in influencing health behavior change. The studies mentioned above lack a control condition in which all design features are present except the mirror to study the direct impact of seeing one’s self-reflection.

A recent study by Jung et al [14] used a projector and a mirror to show participants their bodies with x-ray visualization of smoking lungs. A separate condition displayed the same content but on a mannequin. Both conditions were compared to a control, which displayed the information on a screen in 2D. The researchers found that spatial augmented reality increases spatial presence, the perceptual illusion that the real world and the mediated world are “equally present.” In addition, they found that higher levels of spatial presence were associated with a negative emotional change toward cigarettes and cigarette cessation campaign engagement intention. These findings support the idea that displaying the consequences of health behaviors on top of the user’s own body can impact behavior change constructs regarding emotions and intention. Our study aimed to contribute to this line of research by exploring additional behavior change constructs.

One notable finding from Jung et al [14] was that the mannequin condition also resulted in higher levels of spatial presence. However, an analysis comparing emotions and intentions reported for those in the mannequin condition compared to the control was not reported. Gaining a better understanding of how objects that can serve as external self-representations, such as avatars and mannequins, could help develop more feasible design interventions when mapping AR elements directly on the body would be complex.

Yee and Bailenson [28] found that self-representations can help form our behaviors, even when this representation is digital, such as in the case of avatars. Yee and Bailenson [28] call this the Proteus effect and provided support from two experiments. In study one, participants were provided with an avatar that was previously rated as high, medium, or low on an attractiveness scale. They were asked to interact with another character (in a virtual reality environment) after looking at themselves in a mirror. Those in the high attractiveness condition disclosed more information and moved closer to the other character. The second experiment, testing avatar height in an ultimatum game, found that those in the tall condition were more likely to offer an unfair split. Those in the short condition were more likely to accept an unfair split. These findings suggest that augmentations to self-representation, as an avatar, may impact one’s behavior. Fox and Bailenson [29] studied whether vicarious reinforcement with a user’s avatar had an effect on physical exercise. Seeing one’s avatar benefit from exercise behavior and experience consequences from not engaging in the behavior encouraged the observer to engage in the behavior. These results suggest that vicarious reinforcement using avatars may be effective. Based on the studies mentioned above, we expected that the display of health threats on an avatar representation of the self in an AR environment will impact levels of threat severity, susceptibility, fear, and message minimization.

Our study expands on existing research by investigating how health behavior self-focused AR may impact specific predictors of behavioral intentions and what negative implications may exist in regard to fear control responses.

### This Study

In our research, we examined the impact of self-focused AR and vicarious reinforcement on perception and emotion as it relates to hand washing health beliefs and behavioral intentions. Below, we present our hypotheses:

H1: The combination of self-focused AR and vicarious reinforcement will result in higher levels of perceived positive outcome expectancy, perceived threat severity and susceptibility, fear, and message minimization when compared to a control.

H2: Using avatar representations in self-focused AR with vicarious reinforcement will result in higher levels of threat severity, susceptibility, fear, and message minimization compared to a control.

## Methods

**Figure 1:**
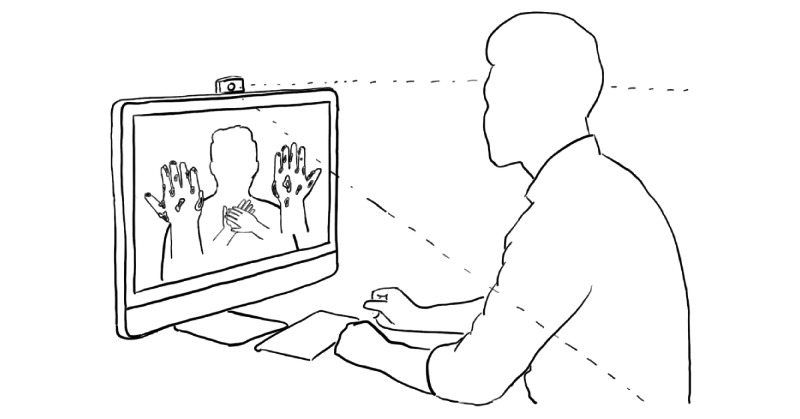
A participant in one of the self-focused augmented reality design groups viewing a reflection of themselves using a video feed from their camera.

**Figure 2:**
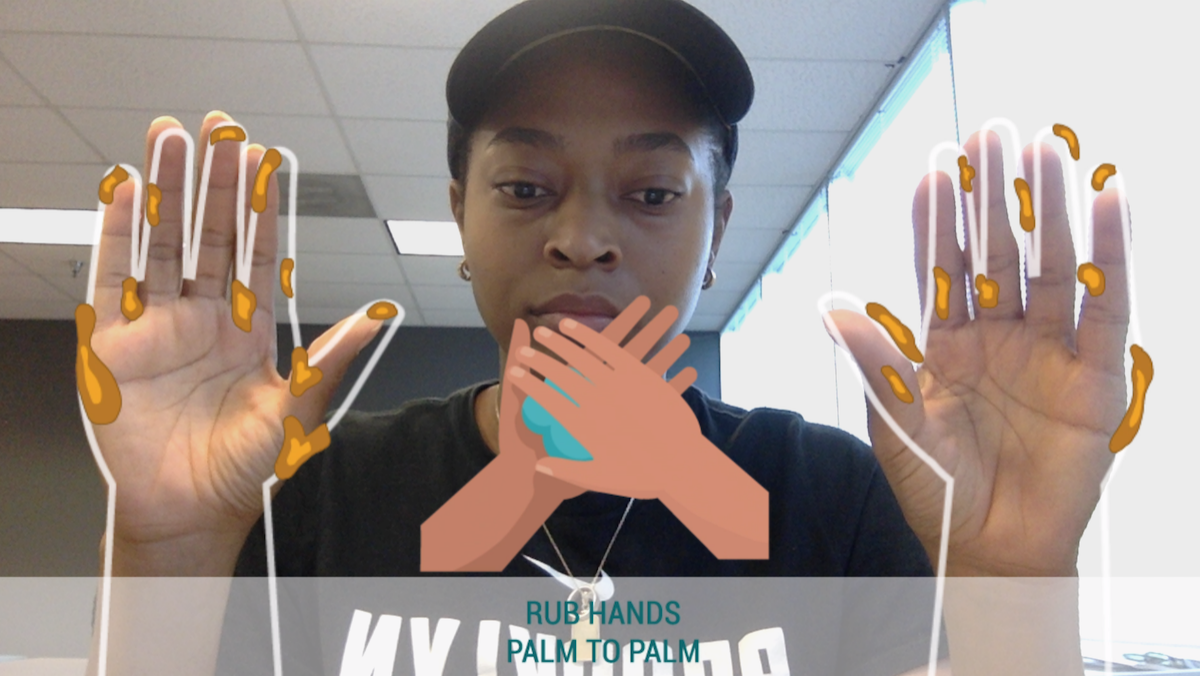
A screenshot from the web application.

**Figure 3:**
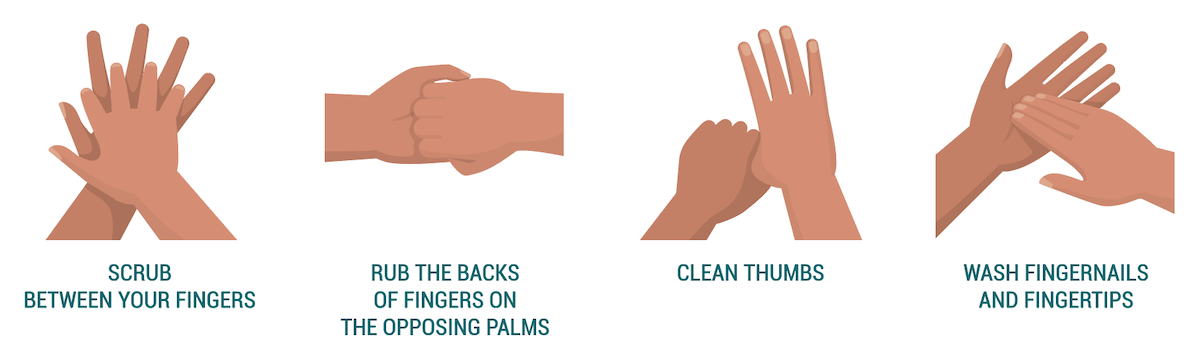
An example of the progression of vectors used in the handwashing animation.

To study the effects of self-focused AR on behavioral intention and perception, we conducted an online experiment. Participants interacted (Figure 1) with a web application (Figure 2) that displayed health information regarding the coronavirus and a hand hygiene animation (Figure 3). Five conditions differed in their inclusion of self-focused attention and vicarious reinforcement.

### Experimental Conditions

We conducted a between-subjects experiment where users interacted with a web application and then responded to an online questionnaire. The study followed a posttest-only control group design to avoid a testing threat to internal validity. The design of the web application differed depending on the intervention condition each participant was randomly allocated to. All five conditions displayed the same information about COVID-19, including how it is spread and preventative measures as described by the US Centers for Disease Control and Prevention (CDC) [17] and the World Health Organization (WHO) [30]. This information was followed by a hand hygiene animation, the display of which differed based on the participant’s assigned condition.

#### Control Condition: No Self-focused AR or Vicarious Reinforcement

**Figure 4:**
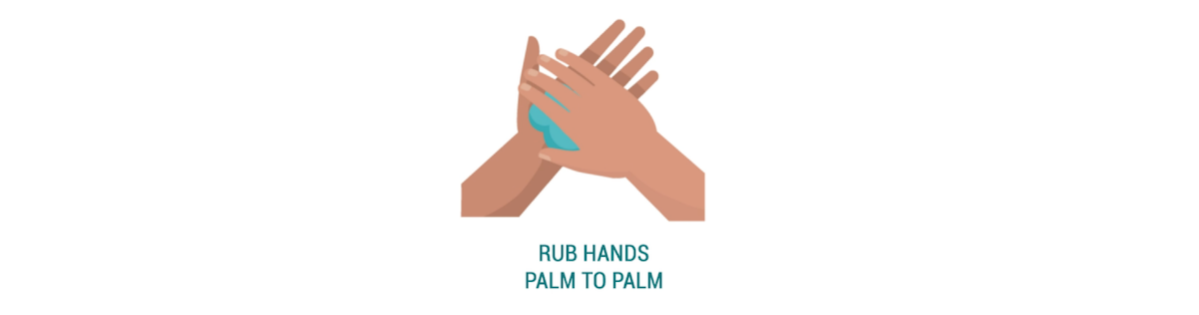
Control condition.

In the control condition (Figure 4), we displayed an animation of a 12-step handwashing technique following standards outlined by the WHO [30], accompanied by captions to describe each movement (“Rub hands palm to palm”).

#### Reinforcement

**Figure 5:**
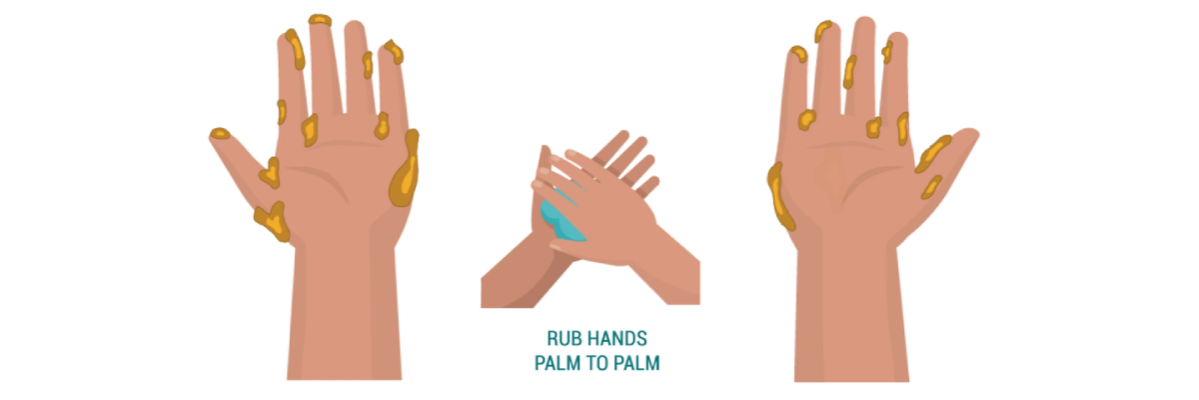
Reinforcement condition.

In the reinforcement condition (Figure 5), the handwashing animation described in the control condition was accompanied by an additional animation showing germs disappearing from a pair of illustrated hands as the handwashing animation progressed. These animations were synced so that the appropriate areas of the hands displayed were affected based on the specific stage of the handwashing animation a viewer was watching. For example, the thumb cleaning animation segment was paired with germs disappearing from the thumbs.

#### Self-focused AR

**Figure 6:**
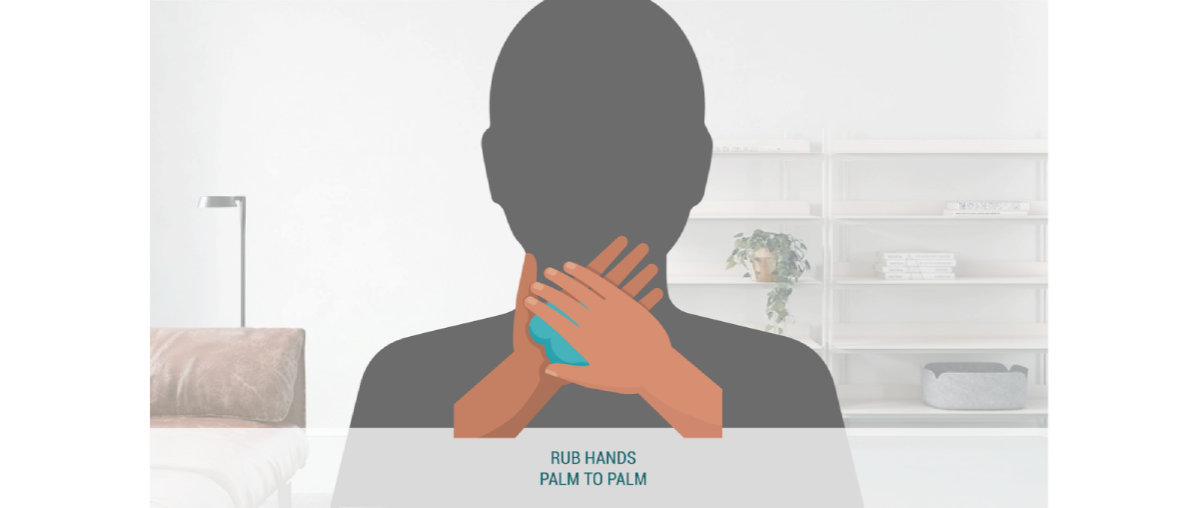
Self-focused augmented reality condition.

The self-focus condition (Figure 6) utilized the participant’s web camera to display their self-reflection, serving as the stimulus for self-focused attention. The handwashing animation was layered on top of the viewer’s reflection. This reflection was shown in real time and was created using the participant’s web camera.

#### Self-focus AR × Reinforcement

**Figure 7:**
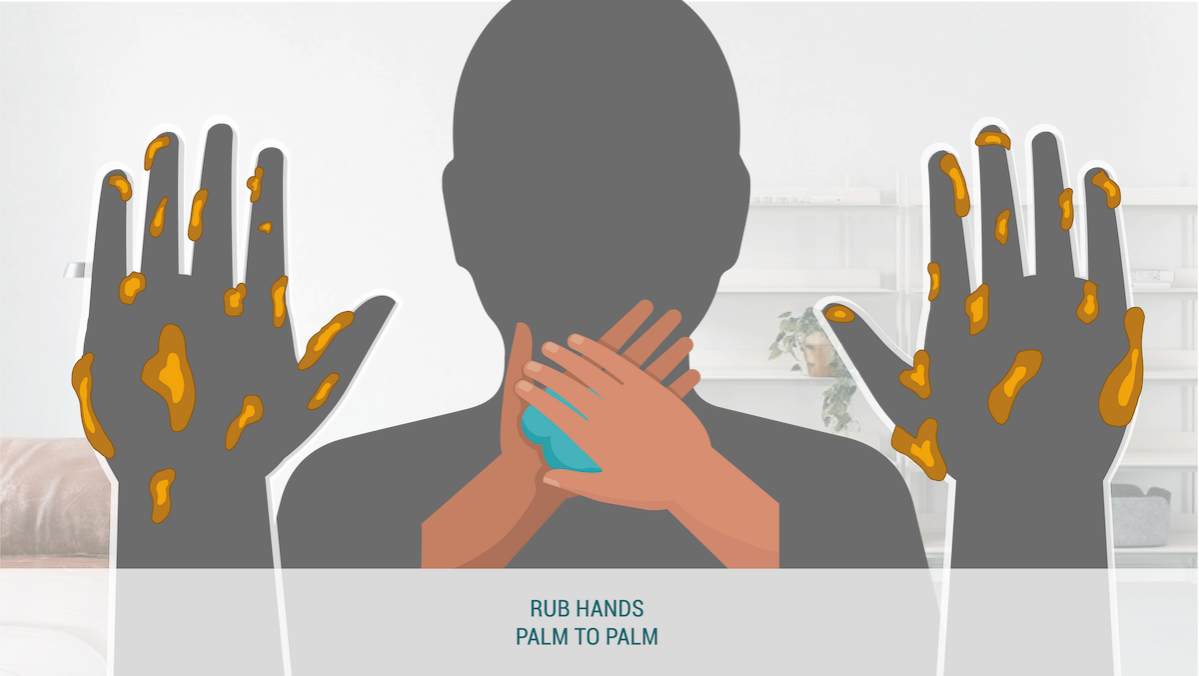
Self-focus augmented reality × reinforcement condition.

The self-focus AR × reinforcement condition (Figure 7) visualized germs directly on the participant’s hands. Instructions at the beginning of the animation directed participants on where to place their hands. The handwashing animation was displayed in between their hands. As the animation progresses, the user saw the germs disappear from the reflection of their own hands.

#### Avatar

**Figure 8:**
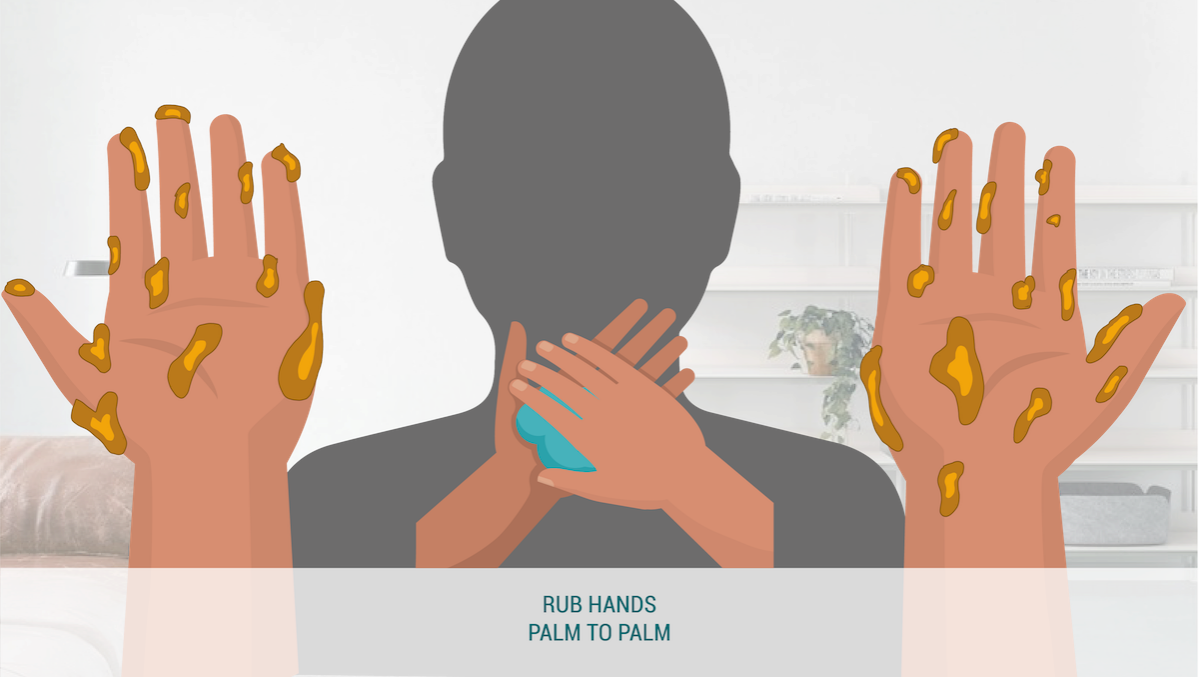
Avatar condition.

In the avatar condition (Figure 8), participants viewed an animation showing germs disappearing from a pair of illustrated hands layered on top of the user’s reflection. These are referred to as avatar hands, as they are meant to represent the user’s hands. The perspective displayed was that which is seen if the individual were to hold up their hands and look at them.

### Participants

Participants were recruited via Prolific (a crowdsourcing platform [31]) and were compensated $2.50 for their time. Participation was limited to those residing in the United States, who spoke fluent English, and were ≥18 years of age. Pilot testing revealed technical challenges that could interfere with the study, mainly involving web camera use verification. To address this, prior to being recruited for the study, Prolific members were invited to a prescreener that verified (1) their access to an acceptable browser for the study (Safari, Chrome, and Firefox) and (2) that camera permissions worked with their technical setup. Prolific IDs for those who passed the screener were collected, and access to the main study was restricted to those IDs.

### Procedure

The experiment took place between August 6-21, 2020. In all five conditions, after receiving consent, we described the experiment as a study on health information presentation and provided instructions to review the information given carefully. In the three self-focused-AR–based conditions, we displayed information on how to set up the web camera for the study.

All conditions provided information about COVID-19. Details focused on how the virus is spread and preventative measures as described by the CDC [17]. Participants next viewed an animation detailing the steps of proper hand hygiene as described by the WHO [30]. This was followed by a questionnaire to collect demographic information and measure health knowledge and perception, which concluded the study.

The questionnaire included questions to check whether the participant is paying attention. Three multiple-choice questions asked the user about the information displayed in the study (eg, What is a recommended preventative measure to reduce the spread of the coronavirus?). To validate that self-focused AR interventions were delivered correctly using the camera, participants in these conditions were informed, prior to the study, that screenshots would be collected randomly throughout the animation. The screenshots were reviewed to ensure that participant’s reflections were displayed to them and that those in the self-focus AR × reinforcement condition had their hands within the view as instructed. Only those who followed the instructions, verified by screenshots, were included in the final data set.

### Variables and Measures

We collected measures of self-reported health beliefs, behavioral intention, and self-reported perceptions of COVID-19 (Table 1), along with demographic data. These measures were adapted from Schwarzer [18] and Li [22]. Items in this study were all measured on a 7-point Likert-type scale ranging from 1 (strongly disagree) to 7 (strongly agree).

Although adapted from previous research, cross loading was a concern due to the rewording of items and the difference in factors present compared to the adapted questionnaires. For example, we added items to measure opinions about perceived threat severity and susceptibility of family and friends. To examine the validity and reliability of our measures, we conducted exploratory and confirmatory factor analysis using a split-sample approach, with one half to develop a model and the other half to validate. Factors loaded as expected (Multimedia Appendix 1).

Data on demographics and the COVID-19 risk of a severe illness of loved ones were collected. Participants were asked to report their age, gender, and the state in which they currently reside. In addition, they were asked to report if they have a family member or friend who is at high risk of severe illness if they are infected with COVID-19. It was noted that one is considered high risk if they are ≥65 years and/or have underlying medical conditions.

**Table 1:** Questionnaire items.

Variable and code		Questionnaire item
**Intention**		
	inte	I intend to wash my hands, as instructed in this study, *on a regular basis.*
**Perceived outcome expectancy**		
	expe1	I believe proper handwashing, as instructed in this study, will help make me *less likely* to get the coronavirus disease (COVID-19).
	expe2	I believe proper handwashing, as instructed in this study, will help *reduce the spread* of the coronavirus disease (COVID-19).
**Fear**		
	fear1	The emotion that I am feeling about the coronavirus (COVID-19) pandemic is: …Frightened
	fear2	…Scared
	fear3	…Anxious
**Message minimization**		
	reac1	To what extent do you feel that *preventative measures messaging*, in your state, regarding the coronavirus disease (COVID-19) is: …Manipulative
	reac2	…Misleading
	reac3	…Distorted
**Perceived** **threat-severity**		
	seve1	I believe that the coronavirus disease (COVID-19) is a serious threat to my personal health.
	seve2	I believe that the coronavirus disease (COVID-19) is a serious threat to my family members (immediate or extended).
	seve3	I believe that the coronavirus disease (COVID-19) is a serious threat to my friends.
	seve4	I believe that the coronavirus disease (COVID-19) is a serious threat to the general public.
**Perceived** **threat-susceptibility**		
	susc1	I am at risk of catching the coronavirus disease (COVID-19).
	susc2	My family (immediate or extended) members are at risk of catching the coronavirus disease (COVID-19).
	susc3	My friends are at risk of catching the coronavirus disease (COVID-19).

### Statistical Analysis

Analysis of our data using histograms and the Shapiro-Wilk test showed that the data were not normally distributed. Shapiro-Wilk *P* values ranged from 6.35e-08 (efficacy) to 1.927e-25 (intention). Therefore, hypothesis testing was conducted using the nonparametric Wilcoxon-Mann-Whitney test, comparing perceived threat severity, susceptibility, outcome expectancy, fear, and message minimization scores between intervention conditions. If condition pairs had the same distribution shape, medians were compared. If the shapes were different, the mean ranks were compared. Additionally, mediation models for dependent variables and design conditions with significant findings were tested. A bootstrapping method using PROCESS macro models 4 and 6 [32], 5000 bootstrap samples, and percentile bootstrap CIs were used. Significance was established at *P*<.05. Statistical analysis was performed using Python (Python Software Foundation) [33], and the pandas (Community) [34] and SciPy (Enthought) libraries [35] were used to conduct the Wilcoxon-Mann-Whitney test. SPSS software (IBM Corp) [36] was resourced, in which the PROCESS macro [32] was implemented to test mediation.

## Results

### Overview

A total of 502 individuals participated in the study. Of this, 335 participants met the attention and screenshot verification checks (see *Procedure* section) and were included in the analysis. Of the 335 participants, 77 were randomly assigned to the control condition, 61 to the self-focused AR condition, 70 to the reinforcement condition, 63 to the self-focus AR × reinforcement condition, and 64 to the avatar condition.

**Figure 9:**
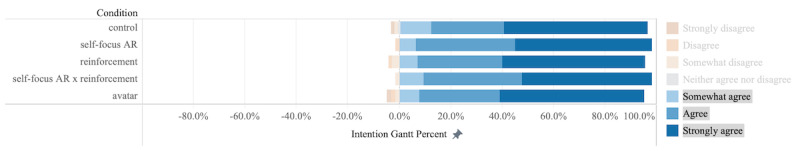
Responses for behavioral intention. Responses are strongly skewed toward higher levels of agreement for all conditions, indicating a ceiling effect. AR: augmented reality.

Although our study focused on predictors of behavioral intentions, we began with results pertaining to intention (Figure 9) to provide context for further discussion. A significant difference between design conditions and the control was not found (self-focused AR: *P*=.42; reinforcement: *P*=.43; self-focus AR × reinforcement: *P*=.41; avatar: *P*=.43).

### Effects of Self-focused AR With Vicarious Reinforcement in Regards to Perceived Fear, Threat Severity, Threat Susceptibility, Outcome Expectancy, and Message Minimization (Hypothesis 1)

**Figure 10:**
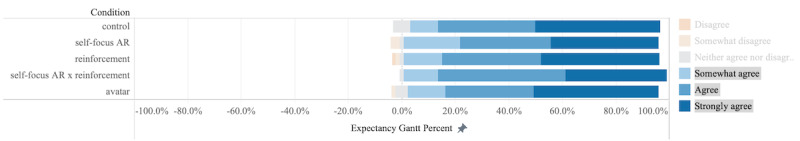
Responses for outcome expectancy. Responses are strongly skewed toward higher levels of agreement for all conditions, indicating a ceiling effect. AR: augmented reality.

Hypothesis 1 proposed that a combination of vicarious reinforcement and self-focused AR would result in higher levels of fear, perceived threat severity, susceptibility, outcome expectancy, and message minimization compared to the control group. Compared with participants in the control (*P*=.43), message minimization scores of those in the self-focus AR × reinforcement condition were not significantly different. This was also the case for outcome expectancy (*P*=.41) and fear (*P*=.23) (Figure 10). However, perceived threat severity and susceptibility had significant findings.

**Figure 11:**
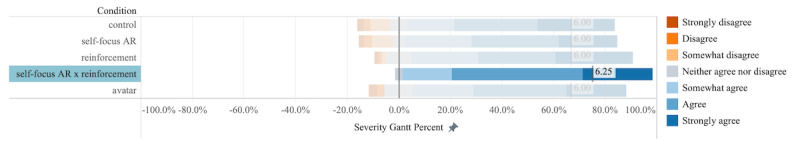
Significantly higher levels of perceived threat severity among participants in the self-focus augmented reality (AR) × reinforcement condition compared to the control condition. No significant differences were found between the control group and the other conditions.

**Figure 12:**
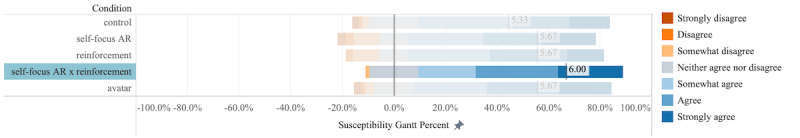
Significantly higher levels of perceived threat susceptibility among participants in the self-focus augmented reality (AR) × reinforcement condition compared to the control condition. No significant differences were found between the control group and the other conditions.

For perceived threat severity, median scores for the self-focus AR × reinforcement and control groups were 6.25 and 6.00, respectively (Figure 11); the two groups’ distributions differed significantly (Mann-Whitney *U*=1983, *P*=.03). Regarding perceived threat susceptibility, median scores for the self-focus AR × reinforcement and control groups were 6.00 and 5.33 (Figure 12); the two groups’ distributions differed significantly (Mann-Whitney *U*=1897.0, *P*=.01). Our results partially supported H1 regarding perceived threat severity and susceptibility; however, we did not find support for outcome expectancy, fear, and message minimization.

Additional analysis revealed that the use of self-focused AR and vicarious reinforcement individually did not impact measured predictors of intention, except for in the case of fear. When compared with participants in the control condition, those in the reinforcement condition (without self-focused AR) did not have a significant difference in medians for outcome expectancy (*P*=.48), threat severity (*P*=.39), susceptibility (*P*=.40), fear (*P*=.10), and message minimization (*P*=.47). Conversely, when compared to the control, those in the self-focused AR condition (without vicarious reinforcement) did not have a significant difference in medians for outcome expectancy (*P*=.26), threat severity (*P*=.21), susceptibility (*P*=.45), and message minimization (*P*=.39). Median scores for the self-focused AR and control groups were 5.0 and 5.3 (*U*=1950.5, *P*=.04).

**Figure 13:**
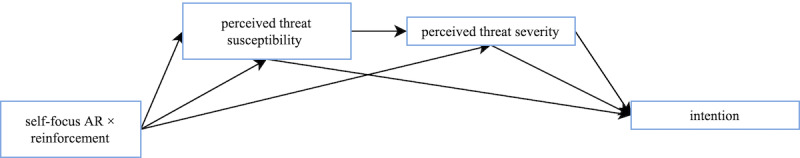
The self-focus augmented reality (AR) × reinforcement, susceptibility, severity, and intention mediation model. The self-focus AR × reinforcement condition resulted in an indirect effect on intention through threat susceptibility and threat severity. This condition also had an indirect effect on perceived threat severity through perceived threat susceptibility.

A mediation model was used to test whether self-focus AR × reinforcement affects behavioral intention through perceived threat susceptibility and perceived threat severity (Figure 13). We found a significant indirect effect of self-focus AR × reinforcement on intention with perceived threat severity as the only mediator (b=.06, 95% CI 0.02-0.12, SE 0.02), but not with perceived threat susceptibility as the only mediator. In addition, a significant indirect effect of self-focus AR × reinforcement on intention was found when both perceived threat susceptibility and threat severity were included as serial mediators (b=.06, 95% CI 0.02-0.12, SE 0.03). These results indicate that although the self-focus AR × reinforcement condition does not directly affect intention in this study, its effect on threat susceptibility and threat severity results in an indirect effect on intention. A separate mediation model was used to investigate whether self-focus AR × reinforcement affects perceived threat severity through perceived threat susceptibility. A significant indirect effect of self-focus AR × reinforcement on perceived threat severity through perceived threat susceptibility was found (b=.26, 95% CI 0.08-0.45, SE 0.09).

**Figure 14:**
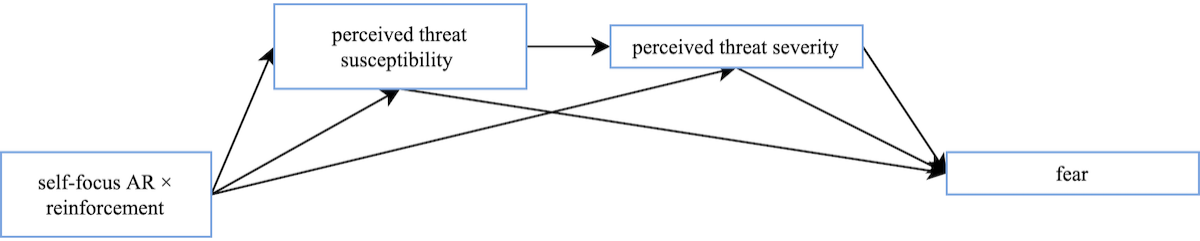
The self-focus augmented reality (AR) × reinforcement, susceptibility, severity, and fear mediation model. The self-focus AR × reinforcement condition resulted in an indirect effect on fear through threat susceptibility and threat severity.

Although fear was not significantly different from the control, we investigated whether fear or message minimization impacted intentions for those in the self-focus AR × reinforcement condition. First, a mediation model was used to test whether self-focus AR × reinforcement affects perceived fear through perceived threat susceptibility and perceived threat severity (Figure 14). A significant indirect effect of self-focus AR × reinforcement on fear was found with perceived threat severity as the only mediator (b=.16, 95% CI 0.05-0.29, SE 0.06) and with perceived threat susceptibility as the only mediator (b=.13, 95% CI 0.04-0.22, SE 0.05). In addition, a significant indirect effect of self-focus AR × reinforcement on fear was found when both perceived threat susceptibility and threat severity were included as serial mediators (b=.15, 95% CI 0.05-0.28, SE 0.06).

Next, a mediation model was used to test whether the self-focus AR × reinforcement condition affects behavioral intention through fear. A significant indirect effect of the self-focus AR × reinforcement condition on intention was found with fear as the mediator (b=.07, 95% CI 0.01-0.16, SE 0.04). An additional model tested whether the self-focus AR × reinforcement condition affects message minimization through fear, threat severity, or threat severity. A significant negative indirect effect of the self-focus AR × reinforcement condition on message minimization was found with severity as the mediator (b=–.07, 95% CI –0.16 to –0.008, SE 0.04. A negative serial mediation effect with susceptibility and severity was also found (b=–.07, 95% CI –0.16 to –0.008, SE 0.04).

### Effects of Using an Avatar on Outcome Expectancy, Fear, Threat Severity, Threat Susceptibility, and Message Minimization (Hypothesis 2)

Hypothesis 2 proposed that combining vicarious reinforcement and self-focused AR while using an avatar would result in higher levels of positive outcome expectancies, fear, perceived threat severity, perceived threat susceptibility, and message minimization when compared to a control. Compared to the control, those in the avatar condition did not have significantly different levels of outcome expectancy (*P*=.42), severity (*P*=.49), susceptibility (*P*=.15), fear (*P*=.23), or message minimization (*P*=.17).

### User Feedback

At the end of the questionnaire, we asked participants to enter optional free-form text about the study. Themes among the responses included impact on knowledge, risk perception formation, and challenges with the self-focus AR × reinforcement condition. Several participants in the reinforcement condition provided generalized statements that the study was educational and helpful. The following comments from the self-focused AR group provided more details.

I thought the handwashing animation together with the illustration of where dirt is cleaned from the hands was very informative.

P1

Some participants learned a new technique or strategy:

I learned some new handwashing techniques! (Particularly, locking your hands together by curling your fingers into each other to get the backs of the fingers).

P2

I hadn’t thought about separately lathering and washing my thumbs.

P3

Comments on personal concerns about risk indicate that future work measuring these variables may consider the time spent around others vs alone and personal risk:

I answered questions knowing that my husband’s and my job allow us to work from home, which decreases our risk significantly, and that most of my family lives in a rural area, also less susceptible to infection.

P4

I know that I take it way more seriously due to the cancer treatment drug I take than most of my friends and peers because if I get it, I am not strong enough to fight it off. I think that factors in way more than friends and family risk, at least for me personally.

P5

Lastly, a few participants in the self-focus AR × reinforcement group expressed confusion about the design. P7, for example, expressed difficulty in positioning themselves on the screen.

It was an interesting survey, but the instruction given for the hand part was kind of hard to complete because the outline of the hands and the picture did not match. However, I tried my best to make it work.

P7

Found the movements in the video hard to follow along with, but I tried my best!

P8

The handwashing directions confused me. At first, I didn’t understand that I wasn’t supposed to mimic the exact instructions.

P9

The responses of P8 and P9 suggest that participants may have practiced along with the video animation. Practicing was not a requirement of participation but appeared to be a trend among those in this condition.

## Discussion

This study explored the impact of self-focus and vicarious reinforcement design interventions on psychological predictors of behavior change during the COVID-19 pandemic. Our results showed that combining self-focused AR with vicarious reinforcement increases perceived threat severity and threat susceptibility and could potentially impact behavioral intentions.

### Behavior Intention and Outcome Expectancy

Our results did not show any direct effects on behavioral intention (Figure 9). Given the severity of COVID-19, we expected these findings to result from a ceiling effect, as similar studies on behavior messaging strategies have found during COVID-19 [37,38]. A surprising finding was the lack of impact of the design interventions on outcome expectancy; however, this also appears to result from a ceiling effect (Figure 10), possibly resulting from the surge of news messaging surrounding hand hygiene’s role in mitigating risk during COVID-19. News segments showed the impact of hand hygiene using blacklights [39] to simulate how hand washing reduces viruses’ presence, which may have helped combat any disbelief. Due to the ceiling effect, our findings require replication studies post–COVID-19 or studies that include intention and outcome expectancy measures less susceptible to the ceiling effect to investigate the impact of self-focused AR and vicarious reinforcement.

### Perceived Threat Severity, Threat Susceptibility, Fear, and Intention

Our findings also revealed interesting relationships between the self-focus AR × reinforcement condition, perceived threat severity, threat susceptibility, fear, and intention. Mediation models showed the self-focus AR × reinforcement condition to positively affect intention and fear through increased perceived threat susceptibility and threat severity. We found self-focus AR × reinforcement to increase perceived threat severity through increased perceived susceptibility. Lastly, we found self-focus AR × reinforcement to indirectly affect intention with fear as the mediator. These results suggest that design strategies that layer a health threat directly on an individual’s reflection may increase one’s perceived threat susceptibility, threat, severity, fear, and indirectly behavioral intention. While such strategies might help meet behavior change design objectives, it is essential to note the potential consequences of designs that increase fear, especially in the context of a public health emergency.

Based on the EPPM, Li [22] tested a model for protective behaviors during a public health emergency with a study during the Ebola outbreak of 2014. The study found perceived threat to have a significant effect on fear and fear controls. Our study partially supports these findings, indicating an impact of threat severity on fear but not a significant positive effect of fear on message minimization (a fear control mechanism). The danger/fear control responses and the impact of self-focused AR likely varies for each health behavior context, as levels of fear will be different for each health threat. While our study did not show adverse effects, researchers and designers should still use caution if utilizing similar design techniques to effect behavior change. More research is needed on the adverse effects of fear concerning triggering fear control mechanisms through design interventions. In addition, increased fear could have mental health implications. Harper et al [20] found fear of COVID-19 to be a positive predictor of behavior change and fear to be correlated with decreased physical and environmental quality of life. Given our findings, designers must investigate the extent to which a design strategy that involves self-focused AR with a health threat increases fear.

### Independent Use of Self-focused Attention and Vicarious Reinforcement

Self-focused AR and vicarious reinforcement embedded as features independently (versus combined) did not show a significant result on any of the tested predictors of behavior change except fear. We provide a few possible explanations for these results. First, regarding mirror self-focus, threat severity, and susceptibility, there may have been too large of a time gap between when participants reviewed the health information provided and when they looked at their self-reflection. In the conditions combining the features, there was content on the screen, reminding participants of the threat at hand. Future work may account for this difference by providing text-based information over one’s reflection. Second, regarding vicarious reinforcement and outcome expectancy, Figure 10 indicates the presence of a ceiling effect. This study may need to be replicated for a different health threat or mitigating behavior that wouldn’t have as many positive, strong outcome expectancy beliefs.

### Limitations and Future Work

It is important to note that this research took place during a long-term global public health emergency with restrictions on lifestyles that can take a while to get adjusted to. Health perceptions related to current circumstances are subject to change throughout the lifecycle of a pandemic. Our findings warrant replication studies that consider changes in severity, government mandates, social perceptions, and the availability and range of tools for risk mitigation (vaccines/medication, personal protective equipment, etc).

The data used in this study are self-reported and susceptible to response biases, specifically social desirability bias. Due to the severity of the pandemic, government mandates, news coverage, and social discussions may have increased the pressure to respond in ways that align with social norms. Future work should aim to use methods to decrease the impact of this limitation.

It is important to note that focusing on individual constructs may create an ineffective design system if the construct only works in combination with other constructs [40]. This should be taken into consideration as self-focused AR is explored in the future, possibly adding measures for other behavior change constructs such as normative beliefs and social facilitation.

While our study indicates that combining self-focused AR with vicarious reinforcement may affect health behavior change by influencing threat severity and susceptibility, we lack a data-driven explanation of why. Future work may benefit from the inclusion of quantitative measures for self-focused attention to compare with severity and susceptibility scores.

Future work may lend itself to developing experimental methods to explore the extent to which self-monitoring, reflective thinking, self-evaluation, and emotion management naturally occur (or do not occur) when using self-focused AR. Conducting these experiments will provide deeper insights into how self-focused AR impacts the psychological mechanism related to behavior change and possibly inspire experiments on how the combination of self-focused attention and other design features could enhance this effect.

According to user feedback, future work should also consider accounting for time spent around others vs alone and personal health when measuring perceived threat severity. Responses also indicate that when measuring perceptions about new messaging, participants should be instructed to respond based on their preferred news source to limit confusion related to the different opinions they hold for individual new sources.

Two comments from the user feedback indicated that participants might have been actually practicing handwashing movements while viewing the animation. During the screenshot verification process, it was noted whether a participant was observed practicing along with the handwashing video. Practicing was not a requirement of participation and was not mentioned in any instructions provided to them. A total of 32 out of 63 participants were observed practicing along with the video in the self-focus AR × reinforcement condition. The self-focused AR and avatar conditions had 7 and 6 individuals observed practicing, respectively. These results cannot be used to make any claims due to the study’s technical setup. Those in the self-focus AR × reinforcement group were instructed to have their hands in view of the camera. Those in other conditions may have practiced off-camera. However, as practicing may affect behavior, this is another potential area for future research.

### Conclusion

As self-focused AR technologies grow in popularity, it is important to understand how such experiences could impact perceptions, emotions, and behavioral intentions. Previous research [5,12-14,28] has explored self-focused AR to varying degrees revealing a potential impact on health behavior. Our study expands upon this work by combining self-focused AR and vicarious reinforcement. Doing so helped to reveal insights about the impact of each feature on perceptions and emotions as they relate to behavior change.

We found that displaying germs disappearing directly from the user’s self-reflection during a handwashing animation will result in higher scores for perceived threat severity and susceptibility when compared to the control or conditions that implemented self-reflection and a display of germs disappearing separately. Increased perceived severity and susceptibility were found to increase behavioral intention. These findings indicate that combining self-focused AR with vicarious reinforcement may be an effective strategy for health communication designers. However, we also voice concern about the possible adverse effects of heightening levels of fear as a design strategy. While our study did not show concerning results, prior research indicates that heightening fear as a health communication strategy can lead to defensive reactions (versus changing behavior) [22,25] and can lower quality of life [20]. We recommend that this be taken into consideration by designers whenever augmenting self-focused attention with a health threat, especially during a public health emergency, as fear may already be at concerningly high levels. Future research should further investigate the role of fear, perceived threat severity, and threat susceptibility when using self-focused AR in health contexts and design strategies for maintaining the well-being of the user while inspiring behavior change.

## Appendix

Multimedia Appendix 1 Factor loadings.

## Abbreviations

AR: augmented reality
CDC: Centers for Disease Control and Prevention
EPPM: Extended Parallel Process Model
WHO: World Health Organization
