# Supplementary material for: Effects of Self-focused Augmented Reality on Health Perceptions During the COVID-19 Pandemic: A Web-Based Between-Subject Experiment
Source: J Med Internet Res. 2021 Jun 29;23(6):e26963. doi: 10.2196/26963 (PMC8244728; doi:10.2196/26963)
Supplement: Multimedia Appendix 1 [file jmir_v23i6e26963_app1.docx]

**Table S1.** Factor Loading

| Items | Efficacy | Expectancy | Fear | Reactance | Severity | Susceptibility |
| --- | --- | --- | --- | --- | --- | --- |
| expe1 | -0.07 | *0.99* | -0.02 | -0.02 | -0.08 | -0.01 |
| expe2 | -0.08 | *0.96* | -0.02 | -0.06 | -0.04 | 0.04 |
| fear1 | 0.03 | -0.05 | *0.98* | 0.02 | -0.03 | 0.02 |
| fear2 | 0.03 | -0.05 | *0.96* | 0.04 | 0.00 | 0.04 |
| fear3 | -0.09 | 0.07 | *0.86* | -0.07 | 0.06 | -0.05 |
| reac2_1 | -0.01 | -0.04 | 0.00 | *0.94* | -0.01 | -0.05 |
| reac2_2 | -0.06 | 0.01 | -0.02 | *0.96* | -0.01 | 0.02 |
| reac2_3 | -0.05 | -0.03 | 0.00 | *0.96* | -0.01 | 0.06 |
| seve1 | 0.04 | -0.05 | 0.05 | 0.05 | *0.98* | -0.10 |
| seve2 | -0.08 | 0.00 | -0.08 | 0.00 | *0.98* | 0.03 |
| seve3 | 0.06 | -0.07 | 0.03 | 0.05 | *0.92* | 0.04 |
| seve4 | -0.07 | -0.01 | 0.03 | -0.15 | *0.81* | 0.05 |
| susc1 | 0.03 | 0.01 | 0.04 | -0.02 | -0.14 | *0.99* |
| susc2 | -0.09 | 0.07 | 0.05 | 0.00 | 0.25 | *0.69* |
| susc3 | 0.07 | -0.03 | -0.06 | 0.05 | 0.07 | *0.93* |

First, the sample was randomly split, and an exploratory factor analysis for half the sample (n = 167) was conducted. The Kaiser-Meyer-Olkin (KMO) value of sampling adequacy was 0.84. Bartlett’s test of sphericity was significant (χ^2^ 5746.04, *P* < .001). The principal factor extraction method was used to extract the factors instead of the maximum likelihood method due to multivariate normality violations. This violation was expected from the participant’s responses due to the strong effect the pandemic could have on health beliefs at the time. An oblique rotation was used because correlations are found between some of the health variables such as severity, expectancy, evaluations, susceptibility, and fear.

A confirmatory factor analysis was conducted on the remaining half of the sample, revealing that the eight-factor model is an adequate fit to the data, with x2= 214.58, x2/df = 2.06 with *P* < .001. Additionally, CFI = 0.96, TLI = 0.95, RMSEA = 0.08. Items with significant loadings (> .40) (Table 2). were averaged to create a score for each factor. For example, Fear is the average of the three fear items.
